# Impact of PAC Fines in Fouling of Polymeric and Ceramic Low-Pressure Membranes for Drinking Water Treatment

**DOI:** 10.3390/membranes6030038

**Published:** 2016-07-07

**Authors:** Laurent Oligny, Pierre R. Bérubé, Benoit Barbeau

**Affiliations:** 1 NSERC-Industrial Chair in Drinking Water Treatment, Department of Civil, Geological and Mining Engineering, Polytechnique Montréal, P.O. Box 6079, Succursale Centre-Ville, Montréal, QC H3C 3A7, Canada; laurent.oligny@veolia.com; 2 Department of Civil Engineering, The University of British Columbia, 6250 Applied Science Lane, Vancouver, BC V6T 1Z4, Canada; berube@mail.ubc.ca

**Keywords:** ceramic membrane, hybrid membrane process, low-pressure membrane, membrane fouling, powdered activated carbon

## Abstract

This study assessed the issue of membrane fouling in a Hybrid Membrane Process (HMP) due to the export of powdered activated carbon (PAC) fines from a pretreatment contactor. Two parallel pilot-scale ceramic and polymeric membranes were studied. Reversible and irreversible foulings were measured following three cleaning procedures: Physical backwashing (BW), chemically enhanced backwashing (CEB) and Clean-in-Place (CIP). The impacts on fouling of membrane type, operation flux increase and the presence/absence of the PAC pretreatment were investigated. Membranes without pretreatment were operated in parallel as a control. In addition, CIP washwaters samples were analyzed to measure organic and inorganic foulants removed from the membranes. It was observed that for the polymeric membranes, fouling generally increased with the presence of the PAC pretreatment because of the export of fines. On the contrary, the ceramic membranes were not significantly impacted by their presence. The analysis of CIP washwaters showed a greater total organic carbon (TOC) content on membranes with a PAC pretreatment while no similar conclusion could be made for inorganic foulants.

## 1. Introduction

The emergence of more restrictive drinking water regulations for the control of resistant microorganisms such as protozoan parasites has favored the use of low-pressure membranes (LPM, i.e., ultrafiltration and microfiltration) as an alternative to the conventional water treatment process (coagulation-flocculation-filtration). Due to the limitation of LPM in addressing the removal of dissolved contaminants (e.g., algae toxins, pesticides, etc.) they are often combined with a pretreatment of powdered activated carbon (PAC).

The use of PAC prior to LPM processes is often performed in combination with coagulation by continuously dosing PAC before a settler or in a direct filtration mode. For such configurations, only a fraction of the activated carbon capacity is used because of the short residence times [1]. In order to reduce operational costs and increase process performance, the combination of a high concentration PAC contactor (several grams per L) with LPM has been developed and is referred to as the Hybrid Membrane Process (HMP). As reviewed by Stoquart et al. (2012), HMP can be divided in two main configurations, where membranes are either immersed or separated from the PAC suspension [2]. While most research to date has focused on the first configuration [3,4,5,6], it has been observed that direct contact of LPMs with aged PAC can lead to excessive irreversible fouling due to modifications to PAC characteristics inside the contactor following the development of biofilm and/or the increase of PAC micro-particles concentration caused by the constant aeration required inside the contactor to prevent PAC settling [3,4,6]. The abrasion produced by the aeration of a high concentration PAC suspension is also a concern for the long-term physical integrity of immersed polymeric hollow fibers [2]. Consequently, separating the membranes from the PAC solution (i.e., contactor) may limit the potential adverse impacts of PAC particles on membrane fouling and integrity. However, this configuration requires an intermediate step to separate and retain the PAC inside the contactor. Micro-screens could potentially be used for such application.

Even if the PAC contactor is separated from the membranes by the use of a separation process such as a micro-strainer, it is expected that carbon fines will be exported from the contactor to the membrane and contribute to membrane fouling [3,7]. Indeed, Khan et al. (2011) noticed that agitation and aeration led to a reduction of PAC particles size distribution inside the contactor. This phenomenon would favor the export of particles having a size lower than the mesh size of the micro-screen; a phenomenon that could induce membrane blocking [8]. In addition, the impact of PAC fines may be largely different from one membrane system to another due to various physical, chemical and operational characteristics. Ceramic membranes are increasingly being researched as an alternative to polymeric membranes [9]. Due to their mechanical resistance, they can be backwashed using a higher backpressure, which might be a favorable option to alleviate the impact of PAC fines fouling.

The role of PAC pretreatment on membrane fouling is complex. On one hand, adsorption of natural organic matter (NOM) onto PAC may decrease membrane fouling due to internal adsorption mechanisms [10,11]. On the other hand, PAC particles may increase cake deposition at the membrane surface [4]. NOM-PAC bonding can contribute to fouling by the formation of a thick cake layer [12,13]. Interestingly, in the absence of foulants, such as NOM, high concentrations of virgin PAC have been reported to result in minor loss in permeability [14].

The objective of HMP is to maintain the PAC within the contactor for a period of time as high as several weeks. Under such operating condition, PAC characteristics are expected to undergo significant changes due, in part, to the formation of biofilm on its surface. Past studies have shown that aged PAC suspensions had different settleability characteristics [15] and propensity to foul UF membranes [6]. Clearly, further studies conducted on the HMP should consider the important role of aged PAC on membrane fouling.

The general objective of the present study was to quantify the impact of a high concentration PAC contactor pretreatment on the fouling of low-pressure membranes. Assays were conducted in parallel using MF ceramic and UF polymeric membranes in order to observe the influence of membrane type/configuration. Ceramic membranes were considered for this application because of their mechanical strength, which enables higher backwash pressures to be considered. The MF ceramic and UF polymeric membranes were selected based on their commercial availability for this particular HMP application. The study was conducted on four parallel industrial pilot membrane systems (MF ceramic or UF polymeric membranes, with and without PAC pretreatment). Total physically irreversible and chemically irreversible foulings were measured for different operating fluxes in order to better distinguish the nature of PAC fouling. Finally, chemical washwaters were analyzed to confirm the foulant characteristics.

## 2. Material and Methods

### 2.1. Source Water

This pilot study was conducted using settled water from the Ste-Rose treatment plant (Laval, QC, Canada). Settled water is produced using Mille-Iles River water treated in a sludge blanket clarifier (SUPERPULSATOR^®^) with the use of alum and activated silica. Settled water characteristics are summarized in Table 1 and are fairly typical of conventionally-treated surface waters (turbidity < 1 NTU, DOC < 3 mg C/L).

### 2.2. Pilot-Scale Membrane Set-up

**Figure 1:**
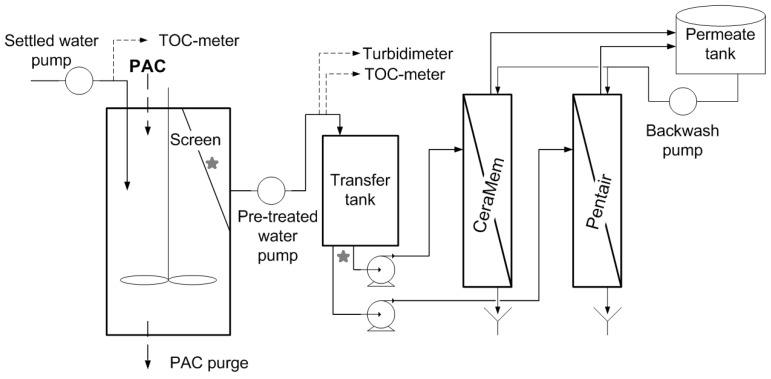
Schematic of the Hybrid Membrane Process (HMP) with powdered activated carbon (PAC) pre-treatment. See Table 2 for design criteria. Stars indicate sampling points used to assess PAC release from the contactor.

The pilot system included two parallel treatment trains, each one feeding two pressurized membranes: ceramic microfiltration (CeraMem^TM^) and polymeric ultrafiltration (Pentair X-Flow, Minneapolis, MN, USA). One treatment train included a pretreatment consisting of a high concentration PAC contactor while the second train had no pretreatment before the membranes and served as a control. Table 2 provides information on the pilot design/operating conditions, while Figure 1 illustrates the treatment train configuration that included the PAC pretreatment. First, settled water was pumped into a stirred activated carbon contactor (CC) in which water was put in contact with a 5 g/L suspension of *d*_50_ = 243 µm PAC (*d*_10_ = 165 µm) (Aquasorb5000, PICA). A fraction of the PAC was purged daily in order to achieve a TOC concentration of less than 2.0 mg C/L in the PAC contactor effluent. Over the course of this project, an average equivalent PAC dosage of 18 mg/L was needed to achieve this objective. TOC concentrations were measured at the influent/effluent of the PAC contactor using an on-line TOC analyzer (Sievers 900, GE Water, Boulder, CO, USA). PAC was maintained inside the contactor and separated from the effluent by an 80-µm micro-strainer (cf. Figure 1). Approximately 0.6% of the PAC particles were below this value. The effluent water was then fed to the membranes. As discussed earlier, an identical treatment train to the one in Figure 1 was operated in parallel without a PAC suspension inside the contactor.

### 2.3. Experimental Design

Experiments were conducted from May to September 2013 during a period when settled water characteristics were fairly stable. Membranes were successively operated at fluxes ranging from 20 to 140 LMH. In order to standardize the experimental conditions, each flux condition was operated until a specific permeate volume of 15,000 L·m^−2^ was filtered. Therefore, assays lasted from a minimum of 4.5 days at 140 LMH to a maximum of 31 days at 20 LMH.

The membranes cleaning procedures differed for both membrane systems (polymeric vs. ceramic) as they were selected to mimic full-scale operation. The experimental plan was therefore not designed to discriminate the impacts of membrane materials bur rather to compare the impacts of differing membrane systems. Firstly, a physical backwash (without chemical addition) was performed every 45 min, which is defined hereafter as one cycle of operation. Secondly, a chemical enhanced backwash (CEB) was performed after 24 cycles of filtration (18 h). Finally, the membranes were washed using a Clean-in-Place (CIP, exhaustive chemical wash) procedure at the end of each flux condition, i.e., after reaching 15,000 L/m^2^. Table 3 summarizes the various cleaning procedures.

### 2.4. Analysis of Fouling Behavior

On-line flow and trans-membrane pressure data were collected to calculate membrane permeability. Permeability data were standardized to a water temperature of 20 °C. Fouling analysis was conducted using two approaches based either on fouling rates or volumetric fouling coefficients.

Fouling coefficients were used to standardize the fouling data based on a volumetric basis, originating from membranes operating at different fluxes. Equation (1) presents the equation used to calculate fouling coefficients (µ expressed in m^2^/m^3^):

(1) $$\frac{Lp_{0}}{Lp_{Vs}}=1+\mu \times Vs$$

where *Lp_0_* and *Lp_Vs_* (at 20 °C) are respectively the initial permeability and the permeability after a filtered volume equal to *Vs*, the latter being defined as the specific filtered volume (m = m^3^/m^2^). Therefore, the fouling coefficient (μ) is obtained by performing a linear regression of the normalized permeability as a function of the specific volume. The fouling coefficient is equivalent to the Unified Membrane Fouling Index and assumes that cake filtration is the main fouling mechanism. Both the fouling rate and coefficient can be used to describe the four types of fouling (described later) investigated in the present study. Results in this paper are mostly presented using fouling coefficients.

In order to test the significance of the observed differences, analysis of variances (ANOVA) or paired t-tests were conducted in STATISTICA 12.0 (Statsoft©, Tulsa, OK, USA). Differences were considered statistically significant at *p* = 0.05 and highly significant at *p* < 0.01.

### 2.5. Types of Fouling

Operating conditions and cleaning procedures were set to allow the investigation of four different types of fouling: total fouling (TF), physically irreversible fouling (PIF), irreversible fouling by Chemical Enhanced Backwash (IF-CEB) and irreversible fouling by a Clean-in-Place procedure (IF-CIP). Equation (1) was adapted for each type of fouling as presented in Equations (2)–(4).

The fouling coefficient for total fouling, which includes both reversible and irreversible fouling, was based on the fouling occurring on a membrane without the effect of backwashing or chemical cleaning during a 45 min filtration cycle and can be expressed using Equation (2):

(2) $$\mu_{TF}=\left(\frac{Lp_{b}}{Lp_{t}}-1\right)\times \frac{1}{Vs_{BW}}$$

where *Lp_b_* is the permeability at the beginning of each cycle of filtration between two backwashing procedures, *b* = [0; # of BW], *Lp_t_* is the permeability values recorded each minute of a 45 min filtration cycle, *t* = [1; 45 min] and *Vs_BW_* is the specific volume filtered between two backwashes.

The fouling coefficient for physically irreversible fouling was based on the permeability loss that was not restored by the hydraulic backwashes conducted after each of the 24 cycles of filtration. It can be expressed using Equation (3)

(3) $$\mu_{PIF}=\left(\frac{Lp_{c}}{Lp_{b}}-1\right)\times \frac{1}{Vs_{CEB}}$$

where *Lp_c_* represents the initial permeability after a chemical cleaning (CEB), *c* = [0; # of CEB], *Lp_b_* is the permeability at the beginning of each cycle of filtration (i.e., between two BW), *b* = [1; # of BW] and *Vs_CEB_* is the specific volume filtered between two CEB procedures.

The fouling coefficient for irreversible fouling by CEB was based on the loss in permeability that was not recovered following this chemical cleaning procedure and can be expressed using Equation (4).

(4) $$\mu_{IF-CEB}=\left(\frac{Lp_{0}}{Lp_{c}}-1\right)\times \frac{1}{Vs_{tot}}$$

where *Lp*_0_ represents the initial permeability at the beginning of each flux assay, *Lp_C_* represents the permeability after each CEB, *c* = [1; # of CEB] and *Vs_tot_* is the total specific volume of the assay (c.f. approx. 15,000 L/m^2^). The total number of data points available to calculate µ*_CEB_* varied from 6 (140 LMH) to 34 (20 LMH).

Irreversible fouling by CIP (IF-CIP) was calculated as a percentage recovery (ρ) of the membrane initial permeability (*Lp_initial_*) using the permeability value after each CIP procedure (*Lp*_0_). Since the membranes were not new at the onset of this study (they had been pretested for a few months on settled waters without PAC), permeability at the start of the 20 LMH assay was used to define initial permeability.

(5) $$\rho =\left(\frac{Lp_{0}}{Lp_{\text{initial}}}\times 100\right)$$

### 2.6. Characterization of Foulants

During each CIP, samples of washwaters were collected after the low and high pH soaking steps. To quantify organic foulants, Total Organic Carbon (TOC) measurements were performed on both acid and hypochlorite cleaning solutions with a combustion-based TOC analyzer (DC-190, Rosemount Dohrmann, Santa Clara, CA, USA). Quantification of metallic/inorganic foulants were conducted using ICP-MS (NexION 300x, PerkinElmer, Waltham, MA, USA) on cleaning solution samples that were first digested to solubilize precipitated inorganic compounds. Acid solutions were digested using HNO_3_ while EDTA was used for bleach solutions.

### 2.7. Measurement of PAC Fines Released from the Carbon Contactor

A test was conducted in order to assess the release of PAC fines from the carbon contactor by collecting samples at different times following one of the periodic PAC dosage in the CC. Two different locations (cf. Figure 1) were sampled: (i) after the CC micro-strainer (i.e., the effluent of the CC contactor) and (ii) on the feed line ahead of a membrane. Particle counts (DPA4000, Brightwell Technologies, Ottawa, ON, CAN) were enumerated at 400X magnification on those samples to evaluate if PAC was effectively exported from the CC to the membranes.

### 2.8. Seasonal Variation of Fouling

In parallel with the pilot plant, a series of lab-scale tests was performed to assess potential seasonal variations in the feedwater fouling capacity. For this purpose, a single hollow-fiber module was built using an UF polymeric membrane module with the same specifications as the pilot UF membranes. Each week, a 2-L settled water sample from the pilot plant influent (i.e., settled water without PAC pretreatment) was filtered at constant pressure (0.9 bar) on the lab-scale membrane module. Temporal flux decline was measured and fouling coefficients (μ) were calculated using the UMFI (Unified Membrane Fouling Index) method [16] described by Equation (1).

## 3. Results

### 3.1. Feedwater Characteristics

General feedwater characteristics of both parallel treatment trains were presented in Table 1. The feedwaters were characterized as having low hardness, alkalinity and pH. Settled waters had an average TOC concentration of 3.03 mg C/L over the course of the study. On one treatment train, the use of the high concentration PAC contactor reduced the TOC concentration to an average of 1.87 mg C/L. However, the turbidity was observed to be higher in the feed water originating from the PAC contactor than in settled waters. The other parameters (pH, alkalinity, hardness) were not impacted by the PAC pretreatment.

### 3.2. Seasonal Fouling Variability

**Figure 2:**
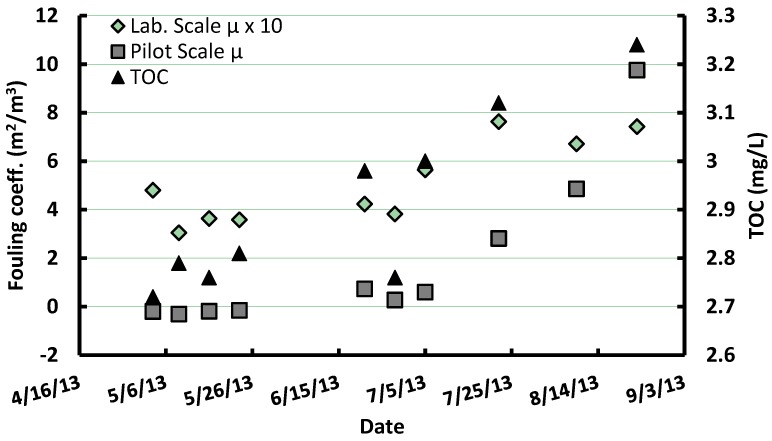
Comparison of lab-scale (1 fiber) and pilot polymeric membrane (w/o pretreatment) total fouling coefficients along with settled water Total Organic Carbon (TOC) concentration variations from May to September 2013.

Figure 2 presents the fouling coefficients obtained at lab-scale using the single hollow fiber module as well as the TOC concentration of the settled feedwater. As a basis of comparison, total fouling coefficients measured on the polymeric pilot membrane (without PAC pretreatment) are also included in Figure 2. During the 19 weeks test period, lab-scale fouling coefficients averaged 0.56 but varied from 0.30 to 0.91 m^2^/m^3^. This variability was related to a temporal trend, which was correlated to TOC variations in the settled feed waters (*r*^2^ = 0.59, *p* = 0.005). A similar correlation was observed when correlating TOC with total fouling coefficients (μ*_TF_*) at pilot-scale for the polymeric membrane without PAC pretreatment (*r*^2^ = 0.70, *p* = 0.005).

### 3.3. Export of PAC Fines from the Carbon Contactor

**Figure 3:**
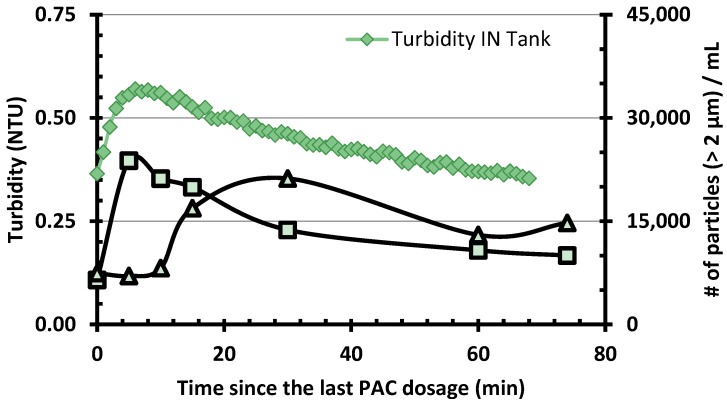
Particle counts and turbidity measured during the PAC export assay. Samples were collected before/after the transfer tank supplying the membranes (see Figure 1).

PAC purges (every 10 to 40 min) and additions (every 25–95 min) in the CC were conducted regularly to keep the PAC age constant. PAC age was adjusted occasionally according to the settled water TOC in order to maintain the TOC effluent concentration under 2 mg C/L. Effluent from the CC transited through a small reservoir (90 L or ≈ 9 min) which was used to feed the pumps delivering waters to the membranes. Figure 3 presents a typical result for the export of PAC fines following one event of PAC addition. Turbidity at the effluent of the carbon contactor feeding the pumping reservoir rose from 0.36 to 0.57 NTU following PAC addition (or 7000–22,000 particles/mL above 2 µm). This effect was observed to last for about one hour. This peak of turbidity induced by PAC was only slightly attenuated by the pumping tank. However, an accumulation of PAC fines at the bottom of this reservoir was noted throughout the study. Nevertheless, Figure 3 indicates that most of the PAC fines did reach the membranes. From the particle size distribution in the tank effluent, one can estimate that each PAC addition (around 10.8 g PAC) in the CC led to the total export of about 210 mg of PAC fines (1.9% of the PAC added) on the membranes (95 mg PAC/m^2^/event). This PAC export is also equivalent to a continuous PAC dosage of 0.35 mg/L.

### 3.4. Fouling Behavior

#### 3.4.1. Typical Fouling Data

**Figure 4:**
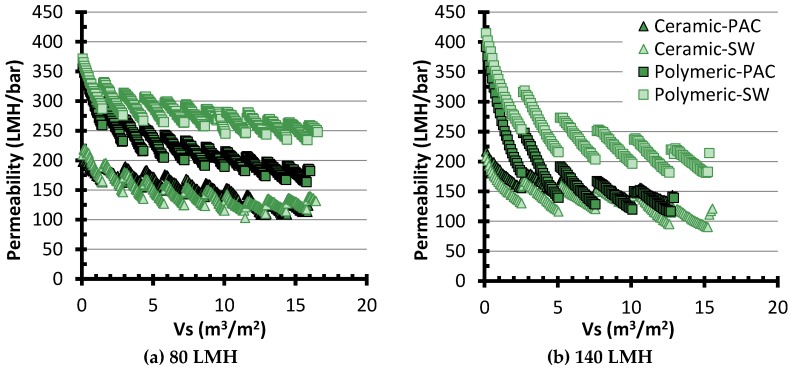
Typical fouling data for assays at (**a**) 80 LMH; and (**b**) 140 LMH. Permeabilities are normalized at 20 °C. SW: Settled Water without PAC pretreatment.

Figure 4 presents typical permeability monitoring data for the assays conducted at 80 and 140 LMH. For one given membrane, each individual slope represents 24 cycles of operation (or 18 h), followed by a CEB, which results in the sudden permeability recovery. From these data, it is apparent that, although the ceramic membrane system had a lower initial permeability, its permeability remained more stable than for the polymeric membrane. Additionally, the polymeric membrane system was negatively impacted by the PAC pretreatment, most likely due to the export of fines. Further analysis of the entire dataset using the concept of fouling coefficient is presented in the following sections.

#### 3.4.2. Total Fouling

**Figure 5:**
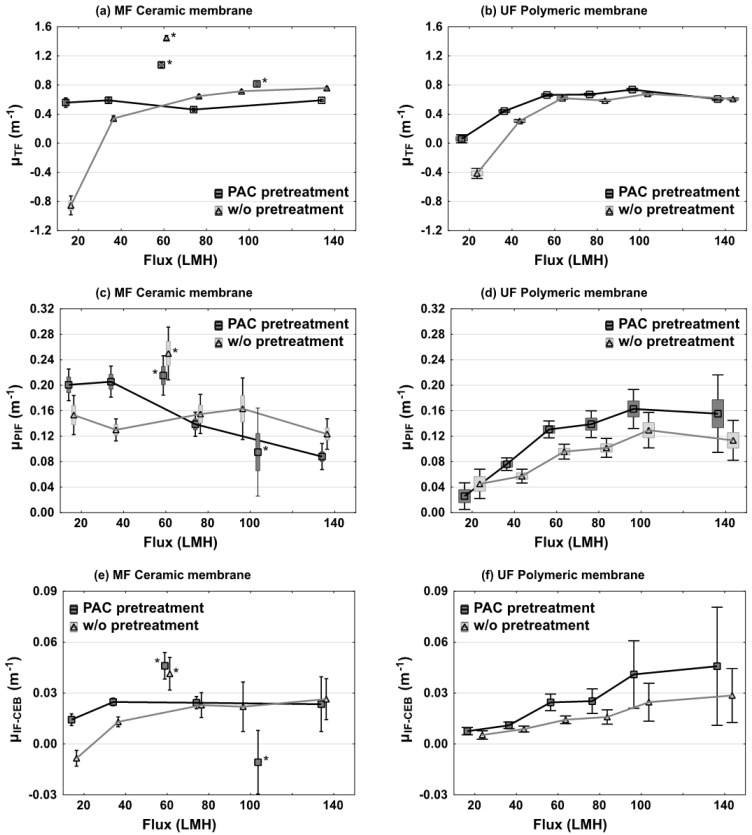
Fouling coefficients of MF ceramic membranes (**a**,**c**,**e**) and UF polymeric membranes (**b**,**d**,**f**) fed with and without PAC pretreated water (black and grey distributions respectively). Markers indicate arithmetic means, boxes represent standard errors and whiskers illustrate the 95% confidence interval. * indicate the three outlier conditions due to improper CIP.

The total fouling coefficients were calculated for the four configurations operated at six increasing fluxes and are summarized in Figure 5a,b. Total fouling was negligible for both membranes while operating at 20 LMH in the absence of PAC. Average fouling coefficients were even negative which implies improved permeability compared to clean water flux, a situation which is in our opinion a reflection of experimental variability and imprecise pressure monitoring at low flux. Three of the experimented conditions (cf. asterisks in Figure 5a,c,e) were considered to be outliers and were excluded from any subsequent statistical analysis. The higher fouling observed at 60 LMH for ceramic membranes was traced back to an improper CIP procedure before this assay (the bleach solution was too diluted), and a feedpump failure occurred at the beginning of the 100 LMH test with PAC pretreatment.

The impact of three different parameters on TF coefficients was evaluated: flux increase, membrane system and PAC pretreatment. Flux was generally found to be the most important factor impacting total fouling coefficients (*p* < 0.01). For three of the four tested conditions, μ*_TF_* increased from 0.3 to 0.7 m^−1^ when the flux was increased. However, the ceramic membrane system receiving a PAC pretreatment was not impacted by a flux increase (*p* > 0.05). The negative effect of higher flux on TF remained stable after a value of 60 LMH for the polymeric membrane and 80 LMH for the ceramic membrane w/o PAC pretreatment. The effect of the PAC pretreatment was found to be significant for the polymeric membrane system (*p* = 0.05). The presence of the PAC pretreatment increased TF by an average of 15%.

#### 3.4.3. Physically Irreversible Fouling

The physically irreversible fouling coefficient (μ*_PIF_*) are presented on Figure 5c,d. Once again, assays with asterisks were not considered in the statistical analysis because of the cleaning and mechanical issues mentioned previously. PIF coefficients were observed to be different between both membrane systems. On the UF polymeric membranes, PIF coefficients progressively increased from 0.03 to 0.12–0.16 m^−1^ as the flux increased from 20 to 140 LMH. For this membrane, PIF coefficients increased on average by 21% (*p* = 0.05) when the PAC pretreatment was applied. For the ceramic membrane system without PAC pretreatment, increasing flux did not impact PIF coefficients, which fluctuated between 0.12 and 0.15 m^−1^. In the presence of a PAC pretreatment, PIF coefficients were even observed to progressively decline from 0.20 to 0.09 m^−1^ when flux increased from 20 to 140 LMH. This behavior was opposite to what was observed for the polymeric membrane system.

#### 3.4.4. Irreversible Fouling by CEB (IF-CEB)

The irreversible fouling by chemical enhanced backwashing (IF-CEB) is illustrated on Figure 5e,f. Values of μ*_IF-CEB_* varied largely, from a low of <0 (no fouling) to a high of 0.045 m^−1^ depending on the experimental conditions. A flux increase generally led to increased fouling, with the exception of the PAC-ceramic system for which IF-CEB was minimally impacted by high flux. For the polymeric membranes, the impact of the PAC pretreatment was more pronounced as it led to an increase in μ*_IF-CEB_* of 57% (*p* = 0.01). For the ceramic membrane system, no significant rise in IF-CEB was noted (*p* = 0.25).

#### 3.4.5. Irreversible Fouling by CIP

The irreversible fouling by CIP was assessed by calculating the permeability recovery after the CIP procedures (Table 4). The baseline (CIP #0) was based on the initial permeability measured before the beginning of the study. For ceramic membranes, the improper CIP procedure after the 40 LMH led to recoveries of only 66%–67% compared to the initial permeability. The CIP problem (pump failure) identified after the 80 LMH assay on the PAC-ceramic membrane also led to a very low recovery (50%). Permeability recoveries by CIP were not statistically different in the absence/presence of a PAC pretreatment (*p* = 0.41) or between the two membrane systems (*p* = 0.17). Overall, the CIP cleaning procedures gave fairly constant recoveries between 80% and 90%. No notable temporal decline in permeability was observed over the course of the study.

#### 3.4.6. Relative Importance of Each Type of Fouling

Table 5 presents an overview of the contribution of three types of fouling to total fouling. Average fouling coefficients of non-outlier conditions are also presented. Irreversible fouling by CIP was excluded from this analysis as no significant trend was detected in the dataset.

For each experimental condition, total fouling was mainly (between 74% and 82%) physically reversible using BW. In the absence of PAC pretreatment, total fouling was not statistically different (*p* < 0.01) for both membrane systems even though the two membranes had largely different MWCOs. Physically reversible fouling was higher for polymeric than ceramic membranes (79%–82% vs. 74%–76%). The lower recovery of the ceramic membrane during BW was compensated through higher recovery during CEB (20%–21% for ceramic vs. 15%–16% for polymeric). The contribution of irreversible fouling by CEB to total fouling was minor (3%–5%) for all four experimented conditions. However, it can be seen that membranes receiving PAC pretreated waters had a higher irreversible fouling by CEB (4.4%–4.7%) than its counterpart without PAC pretreatment (3.3%–3.6%).

### 3.5. Characterization of Irreversible Foulants in CIP Cleaning Solutions

Inorganic and organic irreversible foulants were respectively measured in CIP acid and basic washwaters. Results are presented in Table 6 as the sum of concentrations in both washwaters. For organics, data were normalized as g of TOC per m^2^ of membrane while for inorganics, results are presented as the sum of Al, Ca, Mg and Mn (in g per m^2^ of membrane) expressed in their oxidized (Al(OH)_3_) or precipitated forms (Mg(OH)_2_, CaCO_3_ and MnO_2_). Oxidized iron (Fe(OH)_3_) was also measured but was not considered in the sum of inorganic foulants since a control CIP revealed a background iron contamination that was traced back to a static mixer used to mix the CIP chemicals.

Neglecting iron led to observe that inorganic foulants were essentially composed of aluminum (90%), which was expected as the feedwaters were settled waters pretreated with alum. Overall, the CIP procedures removed 36% more inorganic than organic foulants (if one assumes that NOM foulants are composed of roughly 50% carbon by weight). Differences in chemical washwaters characteristics were observed amongst membranes and pretreatment configurations. Organic foulants were 45% more abundant (*p* < 0.01) on polymeric than ceramic membranes. On the other hand, inorganic foulants were on average 53% more abundant (*p* < 0.01) on ceramic membranes. The more aggressive CIP procedure for ceramic membranes (12 h soaking time versus 6 h for polymeric membranes) may have helped retrieving more inorganic foulants. For both membranes, the presence of a PAC pretreatment slightly increased (*p* < 0.01) the surface concentration of organic foulants. For example, average organic foulants concentrations rose from 0.21 to 0.27 and from 0.31 to 0.39 g C/m^2^ for ceramic and polymeric membranes, respectively. On the contrary, the PAC pretreatment did not make a significant difference on the concentration of inorganic foulants for both membranes (*p* > 0.05). Finally, the impact of flux was not statistically significant.

## 4. Discussion

Two main objectives were targeted in this study: evaluating the impact of a PAC pretreatment on membrane fouling and comparing the behaviors under identical operating conditions of two suitable membranes for the HMP (a MF ceramic vs. a UF polymeric system). Past studies assessing the impact of PAC on membrane fouling have led to contradicting conclusions. Some have noted that PAC reduced fouling [10,11,17] while others concluded that PAC contributed to increase fouling [4,12,18,19]. In the present study, the presence of a PAC pretreatment had an impact on the fouling of the UF polymeric membrane for which TF, PIF and IF-CEB were observed to increase by 15, 21 and 57%, respectively. On the other hand, for the MF ceramic membrane, no fouling type was observed to be impacted by the PAC pretreatment. The goals of the PAC pretreatment were to reduce DOC (mostly for disinfection by-products control) and other trace organic micropollutants. Clearly, a reduction in DOC did not lead to a reduction in membrane fouling. A review performed by Stoquart et al. (2012) suggests that PAC preferably adsorbs NOM fractions, which have a low impact on membrane fouling [2]. In our study, the most severe NOM foulants (humics and biocolloids) had probably already been removed by the alum/settling pretreatment. Therefore, the higher observed fouling in presence of PAC is hypothesized to result from (i) the export of PAC fines which may act as foulants and/or (ii) the secondary interactions of PAC fines with other organic/inorganic foulants.

The export of PAC fines to the membranes was documented in this study, a phenomenon which led to an increase in total fouling on polymeric membranes of 15%. During our study, resistant ceramic membranes were backwashed with a pressure build-up, which was probably more efficient at controlling the effect of PAC fines. Following PAC pretreatment, irreversible fouling by CEB was increased on the polymeric system but not on the ceramic system. The quantification of foulants in the CIP cleaning solutions also demonstrated that more organic foulants were extracted from the membranes fed with PAC pretreated water, an effect which was more pronounced on the polymeric membrane. Foulant-foulant interactions are a complex phenomenon, which is anticipated to be governed by source water characteristics. Zhao et al. (2005) reported that Fe-PAC interactions led to a higher cake resistance than Al-PAC or Ca-PAC interactions [4]. However, even if iron was present in major concentrations in the CIP waters of this study, it is not possible to confirm its role due to its suspected release from external material during acid wash. Nevertheless, our results are in agreement with the work of Londoño (2011) who concluded that irreversible fouling in PAC/UF systems was not the result of pore plugging by PAC fines but rather the result of a modification in the cake layer formation [20].

The impact of flux increase was also assessed in the present study. Previous studies have noted an impact on fouling rate especially while operating above the critical flux [21,22]. While the present study was not focused on determining the membranes critical flux, our results demonstrate that operating below 60–80 LMH was most often beneficial to reduce total and physically irreversible fouling coefficients. In the presence of the PAC pretreatment, flux increase was not a significant factor for the MF ceramic membrane system as opposed to the UF polymeric system. In all cases, the evaluation of flux impact on fouling is biased by the fact that feedwater fouling characteristics changed between flux experiments as evidenced by the lab-scale mini-module. This observation reinforces the need to include a fouling control during membrane study, especially if they are not conducted on parallel treatment trains. The lab-scale mini-module of polymeric membrane proved to be useful to achieve this goal as it behaved similarly to the pilot polymeric membrane.

As a final remark, although PAC was observed to increase fouling on the UF polymeric membrane, it is important to point out that total fouling coefficients were reasonable and could be managed with the use of chemical enhanced backwashes. On the other hand, the superiority of the MF ceramic membrane to mitigate PAC fouling implies the use of more intensive physical backwash and a higher average pressure of operation due to the lower permeability of the MF ceramic monoliths. Future studies should consider alternative options to mitigate the effect of PAC fines on membrane fouling.

## 5. Conclusions

The general objective of this study was to quantify the impact of operating a high concentration PAC contactor on the fouling of low-pressure membrane systems.

Releases of PAC fines from the carbon contactor were measured as equivalent to a continuous dosage of 0.35 mg/L (or 1.9% of the applied PAC dose).

Even though the PAC pretreatment reduced TOC in settled feed water, fouling was observed to increase due to the release of PAC fines.

As opposed to the UF polymeric membrane, fouling on the MF ceramic membrane was not significantly impacted by PAC fines.

Chemical enhanced backwashes and CIP were efficient to recover membrane permeability on both membrane systems.

Acid and caustic/bleach chemical wash revealed more abundant organic deposition on membranes which had undergone a PAC pretreatment. This impact was not significant for inorganic foulants.

Further studies should elucidate the interactions of accumulated PAC fines with other foulants, as well as compare viable options to mitigate their impact on membrane fouling.

## Figures and Tables

**Table 1:** Feed water quality with and w/o powdered activated carbon (PAC) pretreatment.

Parameters	Units	Values	
		w/o PAC Pretreatment	With PAC Pretreatment
Turbidity ^1^	(NTU)	0.2–0.8	0.2–2.0
TOC ^2^	(mg/L)	2.64–3.37 (Avg.: 3.03)	1.19–2.17 (Avg.: 1.87)
pH	–	6.5–7.3	6.5–7.3
Alkalinity	mg CaCO_3_/L	<20	<20
Hardness	mg CaCO_3_/L	25–40	25–40

^1^ Turbidity after membrane filtration was always below 0.07 NTU for both membrane systems; ^2^ TOC removals by both membranes were marginal (≤10%).

**Table 2:** Hybrid Membrane Process (HMP) design parameters and operating conditions.

Parameters	Values			
***PAC Contactor***				
Volume	250 L			
PAC concentrations	Contactor 1:0 g/L (Control) Contactor 2:5 g/L			
Equivalent PAC dosages	Contactor 1:0 mg/L Contactor 2:18 mg/L			
Hydraulic Retention Time	17–32 min depending on water demand			
***Powdered Activated Carbon***				
Type	AquaSorb 5000			
Material	Mineral			
Size	*d*_10_ = 164 µm	*d*_50_ = 243 µm		*d*_90_ = 332 µm
***Membranes***				
Flux investigated	20–40–60–80–100–140 LMH			
Operating mode	*Dead-end*			
Membrane pores	CeraMem: Pentair X-flow:		MF–0.1 µm UF–0.025 µm	
Membrane type	CeraMem: Pentair:		Ceramic (TiO_2_) Polymeric (PES/PVP)	
Membrane area	CeraMem: Pentair:		2.2 m^2^ 3.6 m^2^	
Channel flow dimensions	CeraMem:		2.25 × 2.25 mm^2^	
–	Pentair:		Diameter = 1.5 mm	

**Table 3:** Membranes cleaning procedures.

Types of Cleaning	Values
Physical Backwash	**X-Flow**: 100 LMH Feedwater flow-through for 30 s; Air addition for 10 s; Air/water (100 LMH Permeate backwash) for 20 s; **CeraMem**: 100 LMH Feedwater flow-through for 45 s. 350 LMH Permeate backwash at 45 psi for 30 s.
Chemical Enhanced Backwash	BW at 850 L/h for 45 s; **X-Flow**: Permeate BW at 225 L/h with dosages of 200 mg Cl_2_/L of bleach solution and 500 mg/L of NaOH; 5 min soaking and 60 s Permeate rinse at 900 L/h; **CeraMem**: Permeate BW at 450 L/h with dosage of 500 mg/L of citric acid; 5 min soaking and 60 s permeate rinse at 900 L/h. Same procedure than for X-Flow with initial Permeate BW at 45 psi.
Clean-In-Place	**X-Flow**: Recirculation at 50–100 LMH for 60 min of a citric acid solution (10 g/L) at a pH below 3; 6 h soaking; Recirculation of the same solution at 100–120 LMH for 60 min; Permeate rinse and BW until normal pH recovery; Recirculation at 100–120 LMH for 60 min of a 3 g Cl_2_/L bleach solution (with NaOH at pH above 12) 3 h soaking; Recirculation of the same solution at 100–120 LMH for 60 min; Permeate rinse and BW until normal pH recovery. **CeraMem**: Same procedure than for X-Flow with longer cleaning solutions soaking times of 12 h (citric acid) and 6 h (bleach solution).

**Table 4:** Permeability recovery (%) after the Clean-in-Place (CIP) procedure between each assay conditions.

CIP	Assays (LMH)	Ceramic		Polymeric	
		w/o PAC	PAC	w/o PAC	PAC
0	N.A.	100%	100%	100%	100%
1	After 20	91%	92%	78%	75%
2	After 40	**67%**	**66%**	81%	78%
3	After 60	79%	83%	88%	86%
4	After 80	84%	**50%**	89%	89%
5	After 100	79%	91%	99%	93%
6	After 140	N.A. *	N.A.	N.A.	N.A.

* N.A.: not available. Data in bold indicate CIP for which problems were encountered. See Section 3.4.2 for more details.

**Table 5:** Contributions (%) of fouling types for each membrane.

Types of Fouling	Ceramic				Polymeric			
	Without PAC Pretreatment		With PAC Pretreatment		Without PAC Pretreatment		With PAC Pretreatment	
	(%)	μ (m^−1^)	(%)	μ (m^−1^)	(%)	μ (m^−1^)	(%)	μ (m^−1^)
Total fouling (TF)	100	0.58	100	0.55	100	0.56	100	0.62
Reversible by BW ^1^	76	0.44	74	0.41	82	0.46	79	0.49
Reversible by CEB ^2^	20	0.12	21	0.11	15	0.08	16	0.10
Irreversible by CEB	3.6	0.021	4.4	0.024	3.3	0.018	4.7	0.030

^1^ Reversible by BW = TF–PIF. ^2^ Reversible by CEB = PIF–IF-CEB.

**Table 6:** Organic and inorganic foulant recoveries (in g/m^2^) from CIP washwaters.*

Flux (LMH)	Ceramic				Polymeric			
	Without PAC		With PAC		Without PAC		With PAC	
	Org. g C/m^2^	Inorg. g/m^2^	Org. g C/m^2^	Inorg. g/m^2^	Org. g C/m^2^	Inorg. g/m^2^	Org. g C/m^2^	Inorg. g/m^2^
20	0.21	0.85	0.34	0.98	0.29	0.45	0.32	0.32
40	**0.42**	**0.91**	**0.42**	**0.25**	0.40	0.86	0.43	0.86
60	0.19	0.64	0.27	0.61	0.33	0.05	0.46	0.16
80	0.26	1.01	**0.29**	**0.94**	0.26	0.11	0.33	0.14
100	0.25	0.58	0.18	0.41	0.29	0.12	0.42	0.20
140	0.15	0.31	0.28	0.38	0.27	0.43	0.38	0.39
Avg.	0.21	0.68	0.27	0.59	0.31	0.34	0.39	0.34

* Data in bold indicate CIP for which problems were encountered (see Section 3.4.2). Data in bold were not considered in the statistical analysis.
